# Evolution of Bacterial Cross-Resistance to Lytic Phages and Albicidin Antibiotic

**DOI:** 10.3389/fmicb.2021.658374

**Published:** 2021-06-17

**Authors:** Kaitlyn E. Kortright, Simon Doss-Gollin, Benjamin K. Chan, Paul E. Turner

**Affiliations:** ^1^Program in Microbiology, Yale School of Medicine, New Haven, CT, United States; ^2^Department of Ecology and Evolutionary Biology, Yale University, New Haven, CT, United States

**Keywords:** phage, antibiotic, resistance, evolution, trade-up

## Abstract

Due to concerns over the global increase of antibiotic-resistant bacteria, alternative antibacterial strategies, such as phage therapy, are increasingly being considered. However, evolution of bacterial resistance to new therapeutics is almost a certainty; indeed, it is possible that resistance to alternative treatments might result in an evolved trade-up such as enhanced antibiotic resistance. Here, we hypothesize that selection for *Escherichia coli* bacteria to resist phage T6, phage U115, or albicidin, a DNA gyrase inhibitor, should often result in a pleiotropic trade-up in the form of cross-resistance, because all three antibacterial agents interact with the Tsx porin. Selection imposed by any one of the antibacterials resulted in cross-resistance to all three of them, in each of the 29 spontaneous bacterial mutants examined in this study. Furthermore, cross-resistance did not cause measurable fitness (growth) deficiencies for any of the bacterial mutants, when competed against wild-type *E. coli* in both low-resource and high-resource environments. A combination of whole-genome and targeted sequencing confirmed that mutants differed from wild-type *E. coli via* change(s) in the *tsx* gene. Our results indicate that evolution of cross-resistance occurs frequently in *E. coli* subjected to independent selection by phage T6, phage U115 or albicidin. This study cautions that deployment of new antibacterial therapies such as phage therapy, should be preceded by a thorough investigation of evolutionary consequences of the treatment, to avoid the potential for evolved trade-ups.

## Introduction

As global concerns grow over the widespread emergence of antibiotic-resistant bacteria, attention has increasingly turned to antibiotic alternatives such as phage therapy: the use of bacteria-specific viruses, bacteriophages (phages), to treat bacterial infections. Although phage therapy is frequently seen as a novel medical technology, the approach originated in the early 20th century soon after phages were discovered (d’Herelle, 1917, 1930). However, roughly a decade after the discovery of phages, penicillin was discovered and focus shifted instead to research and deployment of antibiotics (Fleming, 1929). Recently, Western medicine’s interest in phage therapy has resurged as a tool for treating antibiotic resistant infections (Kortright et al., 2019); however, just as antibiotics select for the evolution of antibiotic resistance, phages select for the evolution of phage resistance (Luria and Delbruck, 1943; Azam and Tanji, 2019). To avoid the historical mistakes that resulted in multi- and even pan-drug resistant strains, the evolutionary consequences of phage therapy need to be considered and investigated prior to its widespread use (Cohan et al., 2020).

While the evolution of phage resistance in bacteria is perhaps inevitable, phage therapy strategies can be devised to leverage the evolution of phage resistance as an asset, rather than a limitation (Kortright et al., 2019). In particular, by using lytic phages that interact with bacterial virulence factors and molecules that confer antibiotic resistance, these phages should kill bacteria while selecting for phage resistance that may compromise—or pleiotropically “trade-off” with—virulence or antibiotic-resistance traits (Kortright et al., 2019). Theory, *in vitro* experiments and emergency patient treatment provide evidence that certain phages can direct favorable “trade-off” outcomes, where the therapy kills the target pathogen while selecting for reduced virulence or increased antibiotic sensitivity in the remaining bacterial population (Chan et al., 2018; Gurney et al., 2020; Rodriguez-Gonzalez et al., 2020). This approach exemplifies one goal of evolutionary medicine: applying “evolution thinking” to improve the effectiveness of therapy (Stearns, 2012; Turner, 2016).

However, not all bacterial mutations for phage resistance may constitute a genetic change that alters fitness to create a costly trade-off (Burmeister and Turner, 2020). Instead, the opposite may happen whereby evolution of phage resistance results in an additional fitness gain, also known as a “trade-up.” Indeed, it is possible that the fitness effects of evolved phage resistance might pleiotropically trade-up with virulence or antibiotic resistance (Moulton-Brown and Friman, 2018; Burmeister et al., 2020). To examine this possibility, here we studied the interactions of *Escherichia coli* with two lytic phages and an antibiotic that all require the Tsx porin to enter the cell. Tsx is a substrate-specific outer-membrane porin, which uptakes nucleosides and deoxynucleotides into the periplasmic space (Maier et al., 1988). Phages T6 and U115 are shown to use the Tsx porin as a receptor for binding to *E. coli* (Kortright et al., 2020). Additionally, the Tsx porin uptakes the antibiotic albicidin, a phytotoxic DNA-gyrase inhibitor with clinical potential produced by *Xanthomonas albilineans*, an agriculturally important pathogen that causes leaf scald in sugar cane grasses (Birch and Patil, 1987; Birch et al., 1990; Rott et al., 1996; Hashimi et al., 2007; Kretz et al., 2015; von Eckardstein et al., 2017; Rostock et al., 2018; Hashimi, 2019). Earlier studies examined whether phage T6 or albicidin selected for cross resistance of *E. coli* to the other antibacterials, and found mixed evidence for this idea (Fsihi et al., 1993; Schneider et al., 1993); but these studies looked at small numbers of resistant mutants in a *tsx* knockout background with exogenous expression of Tsx from a plasmid, making it difficult to discern whether or not evolution of cross-resistance would be a general outcome. Here, we isolated larger collections of spontaneous mutants to test the hypothesis that selection for *E. coli* resistance to phage T6, phage U115, or albicidin should tend to produce cross-resistance to the other antibacterial agents, due to converging selection at the *tsx* locus.

Our results confirmed that the predicted pleiotropic trade-ups evolved frequently; selection exerted by any one of the antibacterials led to perfect (100%) cross-resistance of *E. coli* mutants to all three antibacterials. Moreover, we observed that cross-resistance was generally “cost-free” in the absence of phage and antibiotic selection, evidenced by equivalent growth of bacterial mutants relative to their wild-type ancestor in both high- and low-resource laboratory environments. In addition, we used sequence analysis to show that a wide variety of mutations at the *tsx* locus of *E. coli* may govern cross-resistance. Our study suggests that prior characterization of evolutionary consequences of antibacterial treatments, particularly the mechanistic interactions of lytic phages and antibiotics with target bacteria, can be used to inform treatment strategies that potentially avoid the evolution of undesired trade-ups.

## Results

### Phages T6 and U115 Each Select for Cross-Resistance to the Other Phage and to Albicidin

Because phages T6 and U115 both rely on the Tsx porin for cell-binding, we predicted that evolution of resistance to one phage should often lead to cross-resistance against the other phage. To test this idea, we used a classic fluctuation analysis (see “Materials and Methods”) to obtain a collection of spontaneous mutants of *E. coli* that were resistant to each phage individually. We used a paired approach, where each of 10 independently-grown bacterial cultures were used to isolate one T6-resistant and one U115-resistant strain (20 mutants total). Results for efficiency of plaquing (EOP) assays (see “Materials and Methods”) confirmed that growth of phage T6 on each of the 10 T6-resistant mutants (T6R1 through T6R10) was below the limit of detection, compared to normal infectivity of the phage on wild-type bacteria (Supplementary Table 1). Similarly, EOP experiments showed that phage U115 was unable to grow on each of the 10 U115-resistant mutants (U115R1 through U115R10), relative to expected infectivity on the wild-type (Supplementary Table 1). Furthermore, we found positive support for our hypothesis; in all 20 cases, evolved bacterial resistance to phage T6 provided cross-resistance to phage U115 using EOP assays, and *vice versa* (Supplementary Table 1). We concluded that independent selection for *E. coli* resistance to one phage provided cross-resistance to the other phage.

**FIGURE 1:**
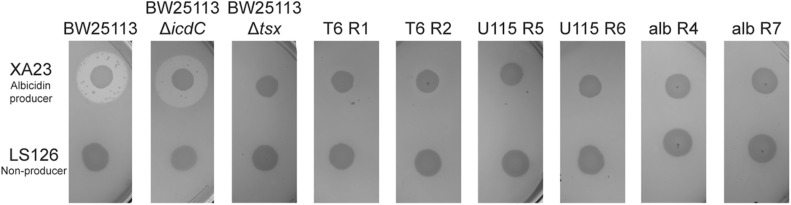
Tests of albicidin resistance for representative spontaneous mutants of *E. coli*, selected for resistance to phage T6, phage U115, or albicidin. Wild-type BW25113 and the positive-control BW25113Δ*icdC* showed inhibited growth on agar plates with albicidin-producing *X. albilineans* (XA23), and normal growth on the non-producer, LS126. In contrast, mutants (T6R1, T6R2, U115R5, U115R6, albR4, and albR7) and negative-control BW25113Δ*tsx* grew equally well in the presence/absence of albicidin-producing bacteria.

We then used antibiotic-resistance assays (see “Materials and Methods”) to test the prediction that evolution of phage resistance should often lead to cross-resistance to the antibiotic albicidin. We first used *E. coli* strains BW25113 and BW25113Δ*icdC* as two positive controls, to confirm that these albicidin-sensitive bacteria fail to form confluent lawns (i.e., they show zones of inhibited growth) when exposed to albicidin-producing *X. albilineans* strain XA23, but grow normally on *X. albilineans* strain LS126 which does not produce albicidin. Results (Figure 1) showed that the controls behaved as expected, with zones of growth inhibition around XA23 indicating sensitivity of both bacterial strains to albicidin. We estimated that wild-type BW25113 had a mean clearing ratio (a ratio of the zone of clearing divided by the area of the *X. albilineans* spot) of 4.88 ± 0.47 s.d. in the presence of XA23 albicidin-producing bacteria, and that BW25113Δ*icdC* bacteria showed a mean clearing ratio of 5.094 ± 0.524 s.d. (Supplementary Figure 1A). As a negative control, the Tsx knockout, BW25113Δ*tsx*, had no zone of inhibited growth on either XA23 or LS126 (Figure 1); in both cases the mean clearing ratio of BW25113Δ*tsx* was 1.0 ± 0.0 s.d. (Supplementary Figure 1A). A test of the hypothesis confirmed our prediction was correct; all 20 phage-resistant mutants showed no zones of growth inhibition around XA23 or LS126 (Figure 1 and Supplementary Figure 1B) and presented mean clearing ratios of 1.0 ± 0.0 s.d. on XA23 and LS126 (Supplementary Figure 1A), indicating that all the phage-resistant mutants were completely resistant to albicidin as well.

### Selection for Albicidin Resistance Confers Cross-Resistance to Phages T6 and U115

We used albicidin selection (see “Materials and Methods”) to isolate nine independent spontaneous mutants of *E. coli* (albR1 through albR9). Using the above-described growth challenges on *X. albilineans* strains XA23 and LS126, our results confirmed that each mutant was albicidin resistant (Figure 1 and Supplementary Figures 1A,B). We then tested whether the albicidin-resistant mutants were cross-resistant to infection with phage T6 and phage U115. Results showed that in all 9 strains tested, acquisition of albicidin resistance conferred cross-resistance to both phages T6 and U115 (Supplementary Table 1). We concluded that the evolution of cross-resistance was absolute in this study system, such that evolution of resistance to one of the three antibacterials provided perfect cross-resistance to all of the selective agents.

### Cross-Resistance to Albicidin and Phages T6 and U115 Is Cost-Free for *Escherichia coli*

**FIGURE 2:**
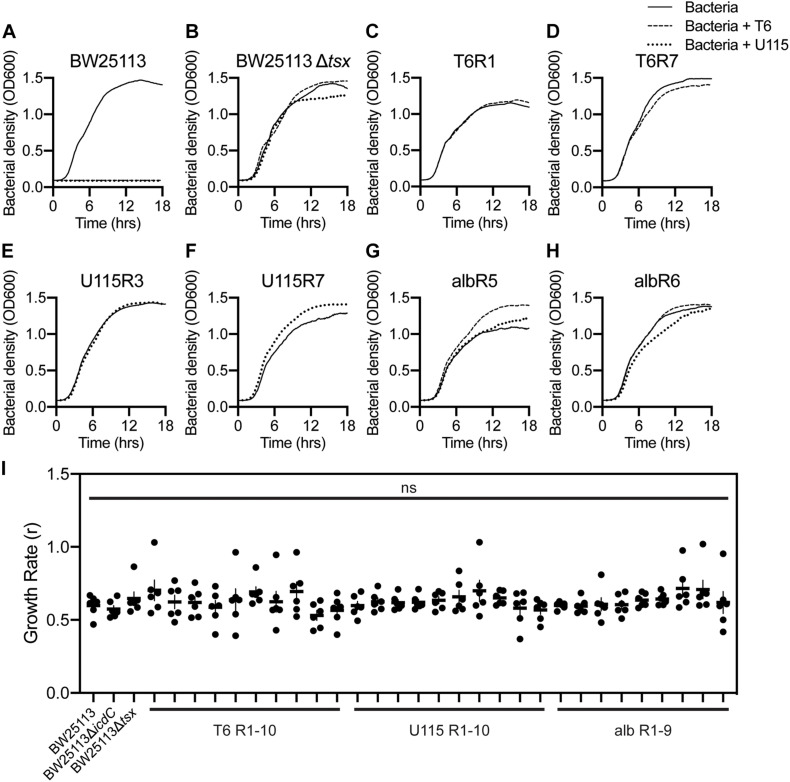
Growth dynamics of representative *E. coli* mutants selected for resistance to phage T6, phage U115, or albicidin, in environments with and without each phage. **(A,B)** Wild-type BW25113 bacteria grew only in phage absence (solid line), whereas Tsx knockout strain BW25113Δ*tsx* grew similarly in phage-free as well as T6 (dashed line) and U115 (dotted line) environments. **(C–H)** Each of the representative resistant mutants grew similarly in the presence/absence of phages T6 and U115, regardless of their prior selection for phage or albicidin resistance. **(I)** Intrinsic growth rate (*r*; proxy for fitness) estimates from growth-curve data [**(A–H)** and Supplementary Figure 2] showed that each strain did not differ statistically from BW25113 (*P* > 0.05; unpaired *t*-tests).

To further examine the evolution of antibacterial cross-resistance, bacterial growth assays (see “Materials and Methods”) were performed with replication (*n* = 3) in LB medium. Controls confirmed that wild-type *E. coli* strain BW25113 showed no discernable growth in the presence of either phage T6 or U115 (Figure 2A), and that growth of the *tsx*-knockout strain, BW25113Δ*tsx*, was similar in the presence and absence of each phage (Figure 2B). In contrast, all 10 of the T6-resistant mutants grew normally in the presence and absence of phage T6 (representative data shown in Figures 2C,D; see Supplementary Figures 2B–I for all results). Similarly, growth of all 10 U115-resistant mutants was unaffected by presence or absence of phage U115 (representative data in Figures 2E,F; see Supplementary Figures 2J–Q for all results). Lastly, the 9 albicidin-resistant mutants were capable of approximately equivalent growth in the presence and absence of either phage T6 or U115 (representative data in Figures 2G,H; see Supplementary Figures 2R–X for all results).

A visual comparison of the growth-curve results suggested that all the resistant mutants grew similarly to wild-type strain BW25113 in the absence of each phage (Figure 2 and Supplementary Figure 2). To examine this outcome more closely, we analyzed the growth data with GrowthCurver (Sprouffske and Wagner, 2016) to estimate intrinsic growth rate (*r*) as a proxy for bacterial fitness (Figure 2I). None of the resistant mutants had an *r* that differed significantly from that of BW25113. These results suggested that there are no appreciable growth costs associated with bacterial evolution of resistance to phage T6, phage U115 and albicidin. While resistance to any of the antibacterials did not affect bacterial fitness under the tested conditions, we could not eliminate the possibility that our growth assays failed to detect minor fitness differences.

**FIGURE 3:**
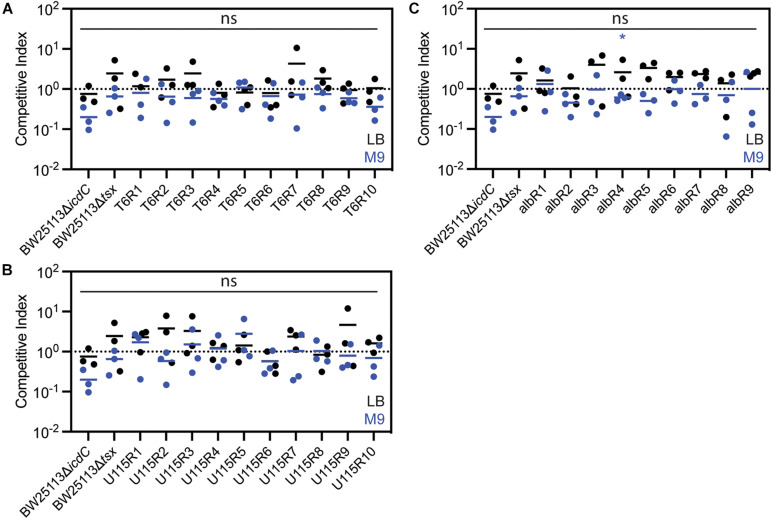
Competition assays in LB media (black) and M9 media (blue) showed that *E. coli* mutants resistant to phage T6 **(A)**, phage U115 **(B)**, or albicidin **(C)** did not measurably differ in fitness, relative to a non-resistant wild-type strain. Each point represents results from independent competition assays (*n* = 3), and statistical significance was determined using a *t*-test comparing competitive index of the resistant mutant relative to the competitive index of BW25113Δ*icdC*. None of the competitive indexes of the resistant mutants were statistically significant in LB media. Competitive indexes of resistant mutants that were statistically significant in M9 media are indicated with * (*P* < 0.05, unpaired *t*-test).

Thus, we conducted additional experiments, in an attempt to measure more subtle fitness differences among bacterial strains. To do so, we performed replicated (*n* = 3) competition assays (see “Materials and Methods”) for each test strain to gauge its fitness relative to a genetically-marked wild-type strain (BW25113Δ*icdC*) in the absence of phage and antibiotic selection. Competitive indexes (CIs) were calculated as the ratio of resistant-mutant colony forming units (CFU) to CFU of the common competitor strain, BW25113Δ*icdC*, to the ratio of BW25113Δ*icdC* CFU to wild-type, BW25113, CFU. Each ratio was normalized by the starting ratio of each competing strain. Results showed no significant differences in CIs of the resistant mutants as compared to the common competitor when competed in a resource-rich complex LB medium for 72 h (Figure 3). Furthermore, when competed in a resource-poor (minimal glucose) defined M9 medium for 24 h (Figure 3), all of the resistant mutants have CIs higher than the CI of BW25113Δ*icdC* with albR4 having the only statistically significant CI. Therefore, it appeared that all T6-resistant, U115-resistant and albicidin-resistant mutants suffered no growth deficits, compared to wild-type bacteria, in the absence of phage and antibiotic selection.

### All Phage-Resistant and Albicidin-Resistant Mutants Have Mutations in *tsx*

**FIGURE 4:**
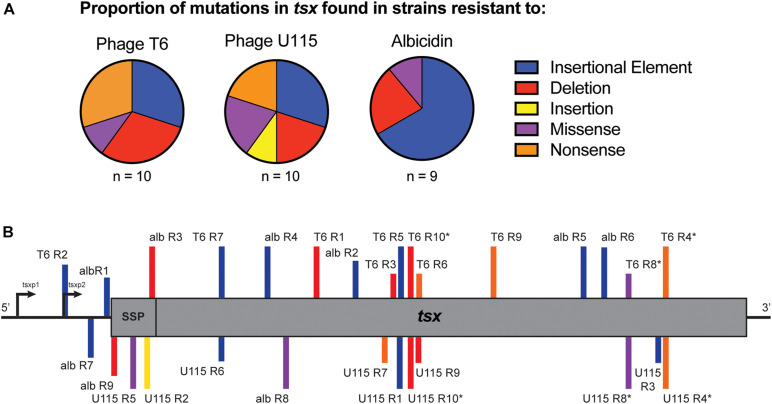
**(A)** Proportions of *tsx* mutations observed in sets of *E. coli* strains, selected for spontaneous resistance to either phage T6, phage U115 or albicidin. Mutations were due to insertion sequence elements (blue), deletions (red), insertions (yellow), missense mutations (purple) and nonsense mutations (orange). **(B)** Locations of *tsx* mutations for each resistant mutant (SSP, signal sequence peptide; *, indicates paired mutants).

Owing to the importance of the Tsx porin in the interactions of all three antibacterials with cells, evolution of *E. coli* cross-resistance suggested that genetic changes in the *tsx* gene were likely involved. To examine this idea, we conducted whole genome sequencing (WGS) of all 29 strains. While some strains showed some single nucleotide variants (SNVs) and short insertions or deletions (indels) in *tsx*, many strains had no observable mutations in any genes using multiple variant calling pipelines including GATK (Van der Auwera et al., 2013) and breseq (Deatherage and Barrick, 2014). While WGS and variant calling pipelines are amenable for detecting SNVs and short indels, structural variants (SVs), including movement of transposable elements, are not easily detected (Ewing, 2015). Therefore, we conducted targeted Sanger sequencing of *tsx* in all 29 resistant mutants. As expected, results for the wild-type showed no mutations in *tsx*; however, mutations in *tsx* were identified in all 29 resistant mutants. For the 10 T6-resistant strains we observed the following mutations in the *tsx* gene: three insertion sequence (IS) elements, three deletions, three nonsense mutations and one missense mutation (Figure 4A and Table 1). For the 10 U115-resistant strains, we documented the following in *tsx*: four IS elements, two deletions, one insertion, two missense mutations, and two nonsense mutations (Figure 4A and Table 1). Interestingly, of the 10 pairs of phage-resistant mutants isolated from the same parent culture, only three pairs (T6R4/U115R4; T6R8/U115R8; T6R10/U115R10) showed identical mutations in *tsx* (Figure 4B and Table 1). For the nine albicidin-resistant mutants we observed six IS elements, two deletions, and one missense mutation in *tsx* (Figure 4A). Of the 29 mutations observed, three were in the promoter (*tsxp2*) region and four were in the signal sequence peptide of Tsx (Figure 4B and Table 1). In addition, our data showed that eight of the 29 mutations in *tsx* clustered within the region spanning base pairs 375–435 (Figure 4B).

**TABLE 1:** Putative resistance mutations in the *tsx* gene, for each *E. coli* mutant selected for spontaneous resistance to either phage T6, phage U115, or albicidin.

Strains	Mutation type	Alteration in *tsx*	Alteration in Tsx
T6 R1	Deletion	deletion of 291-321	E79Stop
T6 R2	Insertional Element	IS2 at -75	promoter region
T6 R3	Deletion	deletion of 402-733	
T6 R4	Nonsense	G773A *	W236Stop
T6 R5	Insertional Element	IS2 at 403	
T6 R6	Nonsense	G435A	W124Stop
T6 R7	Insertional Element	IS2 at 165	IS2
T6 R8	Missense	T725G *	L220R
T6 R9	Nonsense	G535T	E157Stop
T6 R10	Deletion	deletion of 410-420 *	R146Stop
U115 R1	Insertional Element	IS3 at 400	
U115 R2	Insertion	insertion (1bp) at 53	signal peptide; E24Stop
U115 R3	Insertional Element	IS1 at 774	
U115 R4	Nonsense	G773A *	W236Stop
U115 R5	Missense	T31A	signal peptide; V11E
U115 R6	Insertional Element	IS2 at 161	
U115 R7	Nonsense	C375G	Y103Stop
U115 R8	Missense	T725G *	L220R
U115 R9	Deletion	deletion of 429-435	L135Stop
U115 R10	Deletion	deletion of 410-420 *	R146Stop
alb R1	Insertional Element	IS 1 at -3	promoter region
alb R2	Insertional Element	IS1 at 344	
alb R3	Deletion	deletion of 64	signal peptide; L89Stop
alb R4	Insertional Element	IS5 at 116	
alb R5	Insertional Element	IS2 at 665	
alb R6	Insertional Element	IS1 at 692	
alb R7	Insertional Element	IS1 at -16	promoter region
alb R8	Missense	G161C	R32P
alb R9	Deletion	deletion of 4	signal peptide; L89Stop

* Indicates paired phage resistant mutants with the same mutation.

**FIGURE 5:**
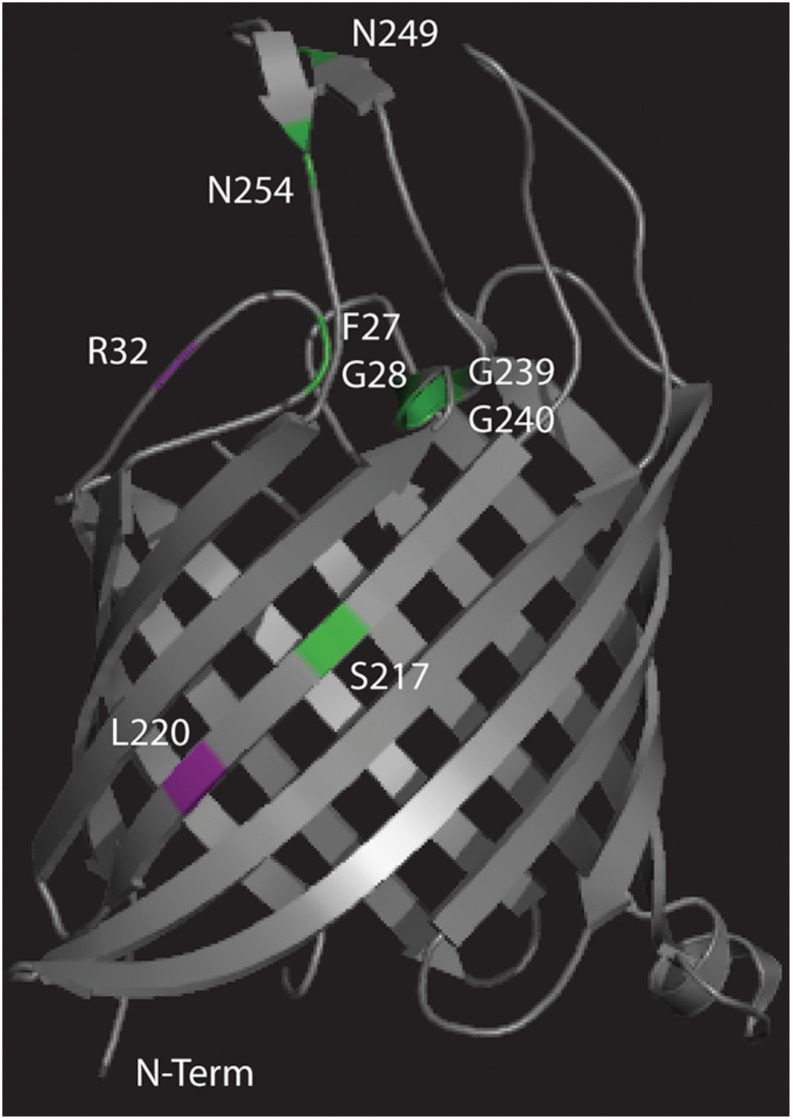
Crystal structure of Tsx (Ye and van den Berg, 2004) with the locations of the missense mutations observed in this study mapped in purple and the locations of missense mutations observed in previous studies (Fsihi et al., 1993; Schneider et al., 1993) mapped in green.

The different missense mutations observed in this experiment were mapped (Figure 5) onto the crystal structure of Tsx (Ye and van den Berg, 2004) along with previously observed missense mutations (Figure 5 and Supplementary Table 2) that conferred resistance to either phage T6 or albicidin (Fsihi et al., 1993; Schneider et al., 1993). Mutant albR8 had a missense mutation R32P in the same surface exposed loop as three missense mutations (F27L, G28R, and G28E) observed by Fsihi et al. (1993) in mutants that were selected for albicidin resistance (Figure 5). Paired mutants T6R8 and U115R8 had the same missense mutation, L220R, in a beta strand that was in the region of a missense mutation (S217R) observed by Fsihi et al. (1993) in another mutant selected for resistance to albicidin (Figure 5). The third missense mutation observed in this study (V11E) in U115R5 is in the N-terminal signal peptide (Table 1).

While it is likely that these mutations are conferring resistance to phage T6, phage U115 and albicidin, this was not experimentally confirmed *via* recombinant genetics because the work fell outside the scope of the current study.

## Discussion

The phage life cycle relies on surface-exposed molecules to initiate infection, and on bacterial metabolism to replicate inside cells. Since phages T6 and U115 both require Tsx as a primary receptor, it is not surprising that spontaneous mutants to one phage confer resistance to the other phage (Kortright et al., 2020). However, it is interesting that all 20 phage resistant mutants are also cross-resistant to albicidin and that all nine albicidin resistant mutants are cross-resistant to both phages. It was previously observed that T6-resistant mutants maintained their sensitivity to colicin-K, another antibiotic that enters *via* the Tsx porin (Schneider et al., 1993). While all three antibacterials used in the current study require Tsx as a receptor, it is unclear whether replication of phages T6 and U115 and the mechanism of action of albicidin also converge on the same gene to confer resistance. Albicidin is a DNA gyrase inhibitor that blocks topoisomerase II, an enzyme which cleaves both strands of DNA to modulate supercoiling during DNA replication, gene regulation, and transcription (Hashimi et al., 2007). Phages require the DNA replication machinery of the host bacterial cell to make viral progeny within the cell; however, many phages, including T6, encode their own topoisomerases (Huang et al., 1985). Furthermore, the minimum inhibitory concentrations (MICs) of ciprofloxacin (another DNA gyrase inhibitor) and ampicillin for wild-type BW25113 bacteria and for each of the 29 resistant mutants did not differ statistically (Supplementary Table 3). These observations of unchanged phenotypes imply that selection by phage T6, phage U115, and albicidin seems to evolutionarily converge on the *tsx* locus, rather than involving changes at other loci. Indeed, the results of the targeted sequencing indicated this possibility, because each mutant was observed to undergo a change in the *tsx* gene (i.e., as opposed to no mutation at this locus, suggesting resistance occurred *via* a different mechanism). While the identified mutations were not validated as the causative mutations of resistance, it seems likely that the 29 different mutations found in *tsx* were conferring resistance to phage T6, phage U115, and albicidin. The combination of *tsx* mutations observed in all 29 resistant mutants and the lack of detectable fitness defects in these strains indicate that Tsx may be superfluous for *E. coli* in the laboratory environment regardless of media type (high resource, complex versus low resource, defined).

The 29 observed mutations in *tsx* fall within the promoter region, as well as various other locations along the gene. However, there seems to be a cluster of “hotspots” for mutational changes in the region spanning 332 to 435 base pairs. These residues make up a surface exposed alpha helix and a beta strand that spans the outer membrane of the bacteria (Ye and van den Berg, 2004). All of the mutations in this region were observed in resistant strains resulting from phage selection but not albicidin selection, indicating that perhaps amino acid residues 88 through 123 are likely important for phage binding.

Moreover, of the 10 “paired” phage-resistant mutants, only three of the pairs (T6R4/U115R4; T6R8/U115R8; T6R10/U115R10) showed the same *tsx* mutation. This means that even in a clonal population of *E. coli*, there are often multiple spontaneous mutants with different changes in *tsx*. This finding indicates that the number of resistant mutants observed to arise during fluctuation assays cannot be used to compute a rate of spontaneous mutation; instead, these data should only be used to calculate a rate of spontaneous phenotypic resistance.

Expression and proper localization of Tsx at the outer membrane occurs *via* two promoters and a signal sequence peptide, respectively (Bremer et al., 1990). Minor promoter, *tsxp1*, is repressed by transcription factor DeoR while major promoter, *tsxp2*, is controlled by the cAMP-CRP complex. Of the three mutations that were observed in the promoter region, all three were in the region of tsxp2, the promoter which results in a higher number of *tsx* transcripts. Likely these mutants (T6R2, albR7, and albR1) are not producing Tsx. Similarly, the four mutants with mutations in the signal sequence peptide region (albR9, U115R5, U115R2, and albR3) likely do not properly express Tsx at the outer membrane.

Indeed, it is probable that many of the resistant mutants do not stably express Tsx in the outer membrane. Mutants with an insertional element disrupting *tsx* (T6R2, T6R5, T6R7, U115R1, U115R3, U115R6, albR1, albR2, albR4, albR5, albR6, and albR7) or indels and nonsense mutaitons at the beginning of *tsx* (T6R1, T6R3, T6R6, T6R9, T6R10, U115R7, U115R9, and U115R10) likely do not express Tsx. Furthermore, Tsx is probably not stably expressed at the outer membrane in resistant mutants with nonsense mutations toward the end of the gene (T6R4 and U115R4). However, it is more likely that Tsx is expressed in resistant mutants with missense mutations (T6R8, U115R5, U115R8, and albR8). Interestingly, of the three different missense mutations observed (T6R8 and U115R8 were paired resistant mutants that share the same mutation), two out of three of them were located in regions where previous missense mutations conferring resistance to albicidin—but not to phage T6—had been observed (Fsihi et al., 1993). It is possible that the L220R mutation (T6R8 and U115R8) in one of the membrane-spanning beta strands is sufficient to disrupt the proper folding and insertion of Tsx in the outer membrane. The V11E mutation (U115R5) in the signal sequence likely disrupts the proper localization of Tsx. The third missense mutation, R32P (albR8), is in a surface exposed loop. Since this mutant was resistant to albicidin, phage T6 and phage U115, it is likely that this particular surface exposed loop is essential for binding of these three antimicrobials; future experiments would be needed to confirm this idea.

It is also interesting that the profile of *tsx* mutations was different for the phage-selected resistant mutants, compared with the antibiotic-selected resistant mutants. There were six albicidin-resistant mutants with an IS element inserted in *tsx*, while three of each of the phage-resistant mutants showed IS-element changes in *tsx.* This observation could be due either to a difference in selection pressures of phages versus antibiotics, or to a difference in the method of selection to amass these mutants. Underlying these possibilities are two opposing ideas. It has been previously assumed that insertion and excision of IS elements are stochastic events. However, recent evidence suggests that IS movement may actually be a form of directed mutagenesis in which IS elements target specific chromosomal loci to reduce stress under certain environmental conditions (Humayun et al., 2017). The isolation of phage-resistant mutants in this study relied on spontaneous mutations accumulated in an overnight culture, while the isolation of antibiotic-resistant mutants allowed for both selection of spontaneous resistant mutants as well as evolution in the presence of albicidin. This difference suggests that the larger proportion of IS element insertions in *tsx* found in the set of albicidin-resistant mutants might be caused by direct targeting of *tsx* by IS elements under stressful conditions. Without further experiments that use the same method to select for spontaneous phage and albicidin resistant mutants, we cannot rule out that a difference in selection pressures between phages and the antibiotic resulted in the skew of IS-element-insertion changes in *tsx* found in the albicidin-resistant mutants.

Finally, it is worth noting that all 29 resistant mutants have gained fitness relative to wild-type in the presence of albicidin or phage, with no discernable growth cost when bacteria are grown without phage and antibiotic selection. Bacteria can utilize nucleosides as a carbon source in environments where carbon is limiting (Tozzi et al., 2006; Mulcahy et al., 2010) and nucleoside-uptake mutations are shown to impair bacterial fitness in a low-carbon environment (Gumpenberger et al., 2016). In this study, we used competition assays to show that phage/antibiotic resistant mutants maintained robust growth in both carbon-rich (LB medium) and carbon-limited (M9 medium) environments. However, it is possible that a cost of antibacterial resistance might occur in different lab-media or other environments not examined here. Antibiotic cross-resistance has extreme clinical relevance as selected resistance to a single antibiotic can also result in evolution of multi-drug resistance. Here, we showed that phage resistance can result in antibiotic resistance, and *vice versa*, at no fitness cost. This highlights a potential pitfall of the future therapeutic use of any antimicrobial whether it be phage or antibiotics. Without a clear understanding of the evolutionary implications of treatment, antimicrobial selection can result in both resistance and cross-resistance at no fitness cost to the bacteria.

## Materials and Methods

### Strains and Culture Conditions

Strains in this study are listed in Supplementary Table 4. *E. coli* strains were cultured for 24 h with shaking incubation at 37°C in lysogeny broth (Luria and Delbruck, 1943). M9 minimal medium (6 g Na_2_HPO_4_, 3 g KH_2_PO_4_, 0.5 g NaCl, 1 g NH_4_Cl, 1 mM MgSO_4_, 0.1 mM CaCl_2_, 0.1% (w/v) glucose per liter) was used for competition assays (see below). The Tsx knockout, BW25113Δ*tsx*, was used as a negative control in many experiments. A pseudogene knockout, BW25113Δ*icdC*, was used as a control for the Kan^*R*^ cassette and as a marked competitor in the competition assays. Where appropriate, LB was supplemented with kanamycin at 30 μg/mL (LB Kan30). *X. albilineans* strains (provided by D. Gabriel, U Florida) were cultured for 48 h with shaking at 30°C in Modified Wilbrinks (MW) medium (Rott et al., 1994). Lysates of phages T6 and U115 were obtained by mixing each phage with *E. coli* strain BW25113 in LB medium and culturing for 24 h at 37°C, followed by filtration (0.22 μm) to remove bacteria.

### Isolation of Phage-Resistant Bacterial Mutants

Ten cultures of *E. coli* strain BW25113 were grown independently as described above, and diluted samples were spread on LB agar (1.5%) plates, pre-saturated with either phage T6 or phage U115. After overnight incubation at 37°C, one colony was chosen randomly from each plate and colony-purified three times. For each of the 20 isolated mutants, phage resistance was confirmed *via* efficiency of plaquing (EOP) assays, which compared plaquing ability of phage T6 or U115 on the test mutant relative to growth on wild-type strain BW25113.

### Isolation of Antibiotic-Resistant Bacterial Mutants

*X. albilineans* strain XA23 was cultured as described above, and a 10 μL sample was spotted onto each of ten Sucrose Peptone agar (SPA) plates that were incubated for 5 days at 30°C (Birch and Patil, 1985). A sample from each of ten independently-grown cultures of BW25113 was then overlaid on one of the SPA plates, and incubated overnight at 37°C. Colonies that appeared in the zone of growth inhibition around the XA23 spot represented spontaneous *E. coli* mutants that were resistant to albicidin. One plate was lost due to contamination; from each of the remaining nine plates, one colony was randomly chosen and colony-purified three times. Albicidin resistance of each mutant was verified by plating a sample of a grown-up culture of the isolate on an XA23 spot, as described above.

### Antibiotic-Resistance Assays

*Xanthomonas albilineans* strains XA23 and LS126 were each cultured for 48 h at 30°C, and their optical densities at wavelength λ = 600 nm (OD_600_) were estimated *via* spectrophotometry. Each culture was then diluted in MW medium, to obtain OD_600_ = 0.25. Then, a 10-μL sample of each diluted culture was spotted on a SPA plate, allowed to dry completely and then incubated for 5 days at 30°C. SPA plates were overlaid with 4 mL of LB top agar (0.75%) with 1 mL of a test *E. coli* strain at OD_600_ = 0.25, and incubated overnight at 37°C. The next day, plates were imaged. Images were quantified using ImageJ, by thresholding until either the *X. albilineans* spots or *E. coli* clearings were outlined and the area within the outline was quantified. The ratio of the area of the zone of clearing to the area of the *X. albilineans* spot was used to define albicidin sensitivity: a ratio of greater than 1.0 indicates albicidin sensitivity, while a ratio of 1.0 indicates albicidin resistance.

Minimum inhibitory concentrations (MICs) of ampicillin and ciprofloxacin were determined using the twofold dilution method (MacLowry et al., 1970).

### Bacterial Growth Curves

*E. coli* bacteria were cultured overnight as described above. Dilutions of a test strain in LB medium were placed in wells of a flat-bottomed 96-well plate, in the absence of phage or mixed with phage at a multiplicity-of-infection (MOI; ratio of phage particles to host cells) of ∼10. Plates were incubated with shaking for 18 h in an automated spectrophotometer at 37°C, and OD_600_ measurements were obtained every 10 min. Growth curve data were fit to a logarithmic curve using GrowthCurver and growth parameters were extracted (Sprouffske and Wagner, 2016). All growth curve data are deposited on Dryad.

### Bacterial Competition Assays

Replicated (*n* = 3) competition assays were conducted in both LB and M9 media by mixing two bacterial strains at a 1:1 initial ratio. Competition assays in LB were serially passaged (1:100 dilution) every 24 h for 72 h total. Competition assays in M9 were measured after 24 h. At the end of each experiment, a diluted sample of each competition was plated on LB agar to measure viable bacterial density (colony-forming units; CFU), and on LB Kan30 agar to estimate the CFU of a Kan^*R*^ competitor; the density of the Kan^*S*^ competitor was estimated by subtraction. Competitive indexes were calculated as the ratio of resistant-mutant CFU to the CFU of BW25113Δ*icdC* divided by the ratio of BW25113Δ*icdC* CFU to BW25113 CFU. Each ratio was normalized by the starting ratio of each competing strain.

### Sequencing

Sequencing libraries were prepared using Illumina’s Nextera prep kit. Whole genome sequencing was done using paired-end 150 bp reads on the Illumina NextSeq platform or paired-end 300bp reads on the Illumina HiSeq platform. For targeted sequencing, primers (5′-CTGTGAAACGAAACATATTTTTG-3′ and 5′-CGTGCTTTTGTTGGC-3′) were designed ∼100 base pairs upstream and downstream of *E. coli* gene *tsx*, and used to amplify *tsx* of each resistant mutant and the ancestral BW25113 strain. Amplicons were Sanger sequenced at the Yale DNA Analysis Facility on Science Hill. Mutations were identified by comparing sequencing reads to *tsx* loci of the reference strain GenBank CP009273.1.

## Data Availability Statement

Sequence data are available at NCBI SRA accession #PRJNA693868. All other data are available on Dryad (https://doi.org/10.5061/dryad.ghx3ffbmn).

## Author Contributions

KK and BC designed study. KK and SD-G conducted the experiments. KK, SD-G, BC, and PT wrote and edited the manuscript. All authors contributed to the article and approved the submitted version.

## Conflict of Interest

PT is a co-founder of Felix Biotechnology Inc., and declares a financial interest in this company that seeks to commercially develop phages for use as therapeutics. PT discloses two provisional patent applications involving phage therapy. The remaining authors declare that the research was conducted in the absence of any commercial or financial relationships that could be construed as a potential conflict of interest.
