# Supplementary material for: Evolution of Bacterial Cross-Resistance to Lytic Phages and Albicidin Antibiotic
Source: Front Microbiol. 2021 Jun 17;12:658374. doi: 10.3389/fmicb.2021.658374 (PMC8245764; doi:10.3389/fmicb.2021.658374)
Supplement: Supplementary file 1 [file Data_Sheet_1.pdf]

Supplementary Material

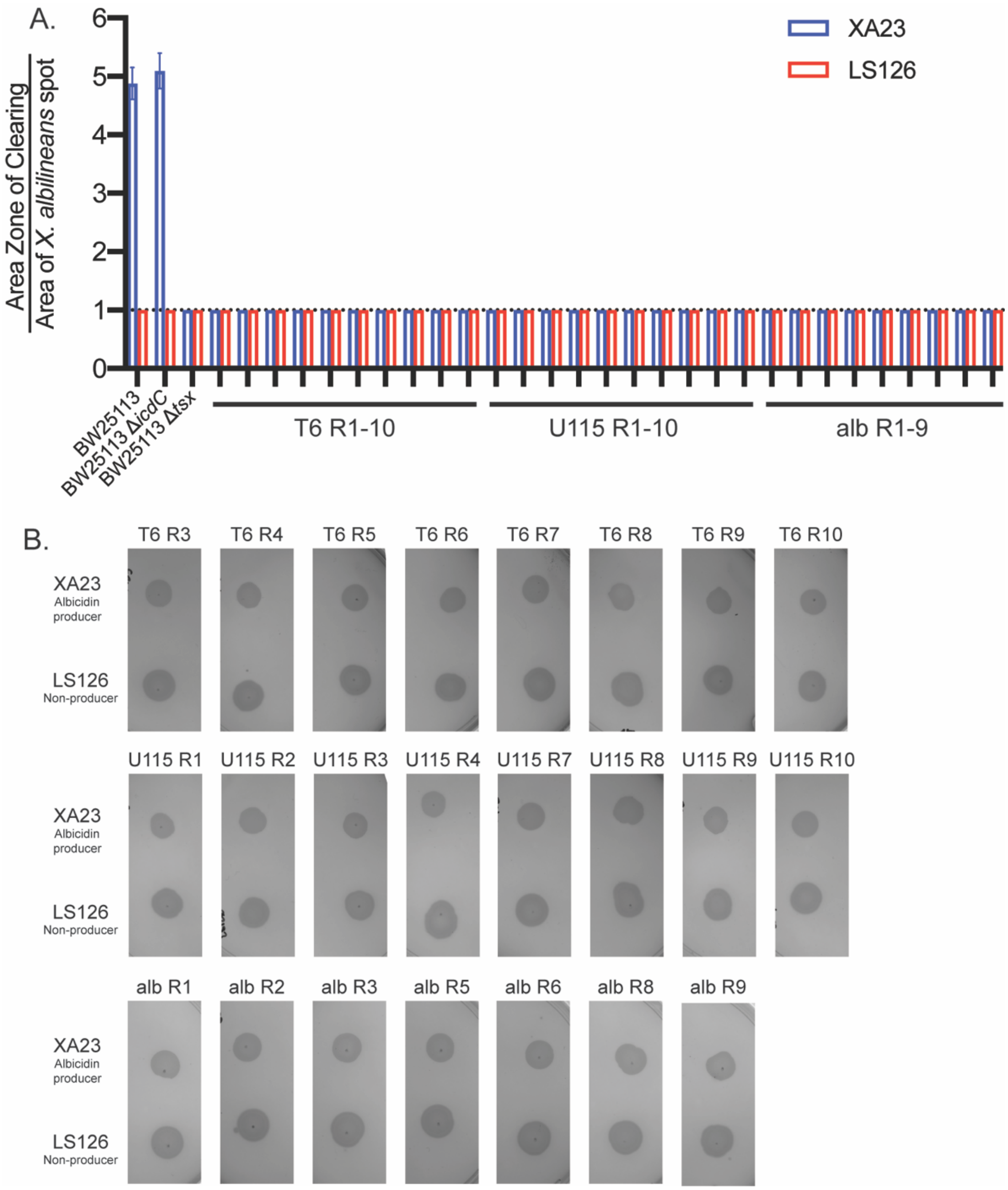

Supplemental Figure S1. Tests of albicidin resistance for spontaneous mutants of *E. coli*, selected for resistance to phage T6, phage U115 or albicidin antibiotic; data are for mutants not described in the main text. **A:** Quantification of albicidin resistance (ratio of cleared zone to area of *X. albilineans* growth) showed that each mutant and the Tsx-knockout strain, BW25113Δ*tsx*, were albicidin-resistant (ratio = 1.0), whereas wildtype BW25113 and positive-control BW25113Δ*icdC* bacteria were

albicidin-sensitive (ratio > 1.0). **B:** In all cases, mutant growth on agar plates was not inhibited by albicidin-producing *X. albilineans* (XA23), and growth was normal on non-producer strain LS126 controls.

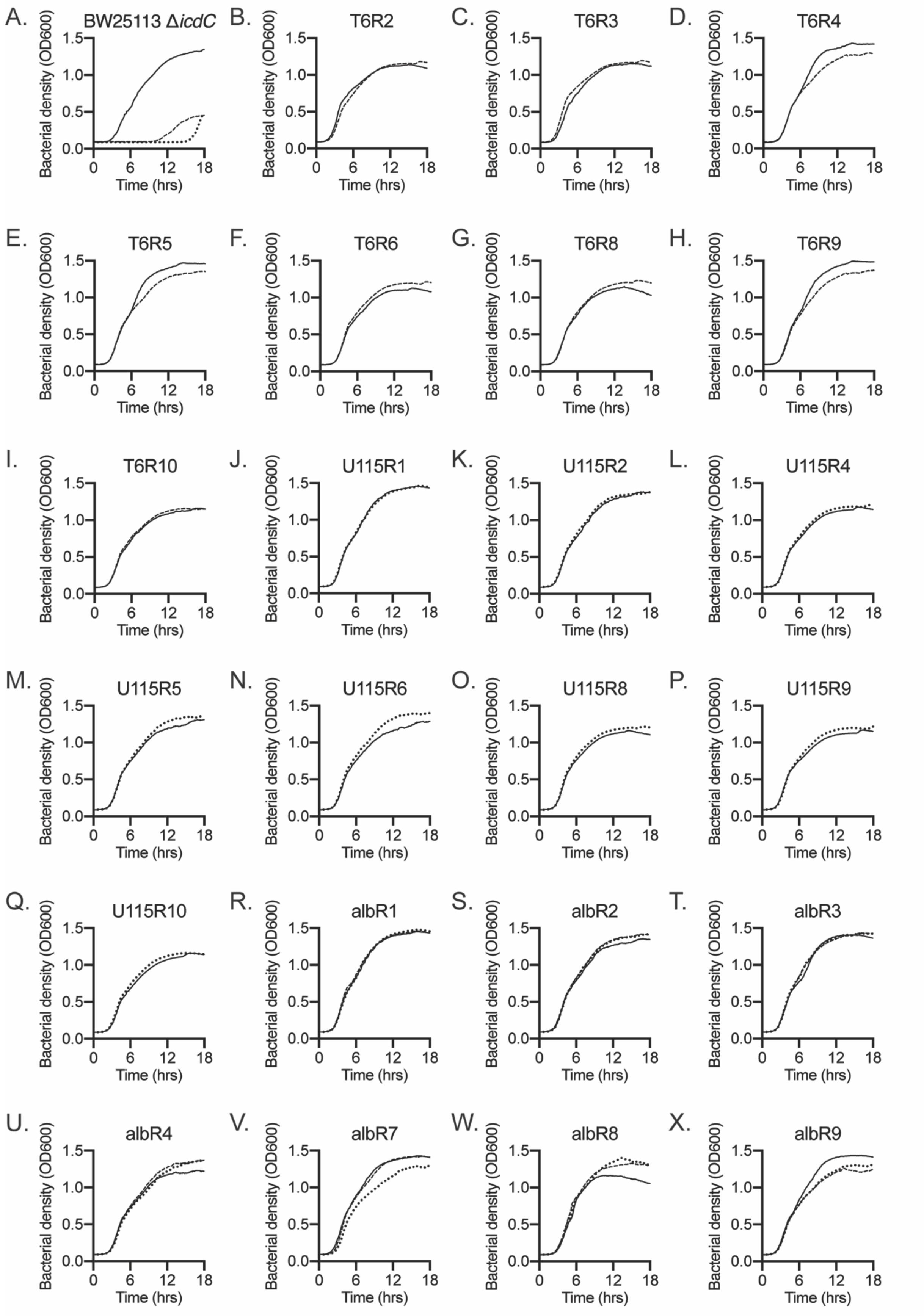
A. BW25113 Δ*icdC*
B. T6R2
C. T6R3
D. T6R4
E. T6R5
F. T6R6
G. T6R8
H. T6R9
I. T6R10
J. U115R1
K. U115R2
L. U115R4
M. U115R5
N. U115R6
O. U115R8
P. U115R9
Q. U115R10
R. albR1
S. albR2
T. albR3
U. albR4
V. albR7
W. albR8
X. albR9
Bacterial density (OD600)
Time (hrs)
0.0
0.5
1.0
1.5
0
6
12
18

Supplemental Figure S2. Growth dynamics of *E. coli* mutants selected for resistance to phage T6, phage U115, or albicidin, in environments with phage T6 (dashed line), with phage U115 (dotted line), and without phages (solid line). **A-X:** In all cases, mutants grew similarly in presence/absence of phages T6 and U115, regardless of their prior selection for phage or albicidin resistance.

Supplemental Table S1. Efficiency of plaquing (EOP) for phages T6 and U115 on challenge strains of *E. coli*, selected for spontaneous resistance to each phage or to albicidin. EOP is defined as phage titer (PFU/mL) on the test strain divided by that on wildtype BW25113; EOP = 0 indicates phage growth on the challenge is below the limit of detection (<100 PFU/mL). Positive (BW25113Δ*icdC*) and negative (BW25113Δ*tsx*) controls confirm expected susceptibility and resistance to each phage in replicate (*n* = 3) assays, whereas, all 29 mutants show cross-resistance to phage infection. Statistical significance (*; $P < 0.05$) indicates result of an unpaired t-test comparing three replicate measures to the EOP on BW25113Δ*icdC*. The '^' indicates a phage with observable turbid clearing of the challenge-bacteria lawn, without making visible plaques.

**Efficiency of Plaquing**	**Phage T6**	**Phage U115**
BW25113 Δ*icdC*	1.11 ± 0.55	1.32 ± 0.16
BW25113 Δ*tsx*	0*	0*
T6 R1	0*	0*
T6 R2	0*	0*
T6 R3	0*	0*
T6 R4	0*	0*
T6 R5	0* ^	0*
T6 R6	0*	0*
T6 R7	0*	0*
T6 R8	0*	0*
T6 R9	0*	0*
T6 R10	0*	0*
U115 R1	0*	0*
U115 R2	0*	0*
U115 R3	0*	0*
U115 R4	0*	0*
U115 R5	0*	0*
U115 R6	0*	0*
U115 R7	0*	0*
U115 R8	0*	0*
U115 R9	0*	0*
U115 R10	0*	0*
alb R1	0*	0*
alb R2	0*	0*
alb R3	0*	0*
alb R4	0*	0*
alb R5	0*	0*
alb R6	0*	0*
alb R7	0*	0*
alb R8	0*	0*
alb R9	0*	0*

Supplemental Table S2. Changes to Tsx and sensitivities to phage T6, albicidin and colicin K for *E. coli* strains that were selected for resistance to phage T6 (Schneider et al., 1993) and resistance to albicidin (Fsihi et al., 1993). Missense mutations are mapped onto the crystal structure of Tsx in green in Figure 5. NA indicates data not available.

			Sensitivity or Resistance to:			
**Strain**	**Alteration in Tsx**	**Selection**	**T6**	**albicidin**	**colicin K**	**Reference**
tsx-504	N249L	T6	R/S	NA	S	Schneider et al., 1993
tsx-505	N254L	T6	R/S	NA	S	Schneider et al., 1993
tsx-506	deletion G239-D244	T6	R/S	NA	R	Schneider et al., 1993
tsx-507	duplication Y229-H237	T6	R/S	NA	S	Schneider et al., 1993
tsx-508	F27L	albicidin	S	R	S	Fsihi et al., 1993
tsx-509	G28R	albicidin	S	R	S	Fsihi et al., 1993
tsx-510	G28E	albicidin	S	R	S	Fsihi et al., 1993
tsx-511	S217R	albicidin	S	R	S	Fsihi et al., 1993
tsx-512	G239D	albicidin	S	R	S	Fsihi et al., 1993
tsx-513	G240D	albicidin	S	R/S	S	Fsihi et al., 1993
tsx-514	duplication G21-N33	albicidin	R/S	R	S	Fsihi et al., 1993

Supplemental Table S3. Estimated minimum inhibitory concentrations of antibiotics ciprofloxacin and ampicillin, capable of inhibiting the growth of each test bacterial strain.

Strain	Ampicillin (μg/mL)	Ciprofloxacin (ng/mL)
BW25113	20 ± 0	11.67 ± 7.64
	20 ± 0	11.67 ± 7.64
	20 ± 0	13.33 ± 5.77
BW25113 Δ*icdC*	20 ± 0	13.33 ± 5.77
BW25113 Δ*tsx*	20 ± 0	6.67 ± 2.89
	26.67 ± 11.55	10 ± 0
T6 R1	20 ± 0	6.67 ± 2.89
T6 R2	20 ± 0	10 ± 0
T6 R3	20 ± 0	8.33 ± 2.89
T6 R4	20 ± 0	8.33 ± 2.89
T6 R5	20 ± 0	10 ± 0
T6 R6	20 ± 0	8.33 ± 2.89
T6 R7	20 ± 0	11.67 ± 7.64
T6 R8	20 ± 0	8.33 ± 2.89
T6 R9	20 ± 0	13.33 ± 5.77
T6 R10	20 ± 0	13.33 ± 5.77
U115 R1	20 ± 0	10 ± 0
U115 R2	20 ± 0	10 ± 0
U115 R3	20 ± 0	10 ± 0
U115 R4	20 ± 0	8.33 ± 2.89
U115 R5	20 ± 0	8.33 ± 2.89
U115 R6	20 ± 0	6.67 ± 2.89
U115 R7	20 ± 0	8.33 ± 2.89
U115 R8	10 ± 0	8.33 ± 2.89
U115 R9	20 ± 0	11.67 ± 7.64
U115 R10	20 ± 0	8.33 ± 2.89
alb R1	20 ± 0	11.67 ± 7.64
alb R2	20 ± 0	13.33 ± 5.77
alb R3	20 ± 0	13.33 ± 5.77
alb R4	20 ± 0	13.33 ± 5.77
alb R5	20 ± 0	8.33 ± 2.89
alb R6	20 ± 0	8.33 ± 2.89
alb R7	20 ± 0	8.33 ± 2.89
alb R8	20 ± 0	13.33 ± 5.77
albR9	20 ± 0	10 ± 0

Supplemental Table S4. Bacterial and phage strains used during this study.

Strains	Species	Antibiotic Resistance Cassette	Reference or Source
BW25113	*E. coli*	-	CGSC
BW25113 Δ*icdC*	*E. coli*	KanR	CGSC
BW25113 Δ*tsx*	*E. coli*	KanR	CGSC
T6 R1	*E. coli*	-	This study
T6 R2	*E. coli*	-	This study
T6 R3	*E. coli*	-	This study
T6 R4	*E. coli*	-	This study
T6 R5	*E. coli*	-	This study
T6 R6	*E. coli*	-	This study
T6 R7	*E. coli*	-	This study
T6 R8	*E. coli*	-	This study
T6 R9	*E. coli*	-	This study
T6 R10	*E. coli*	-	This study
U115 R1	*E. coli*	-	This study
U115 R2	*E. coli*	-	This study
U115 R3	*E. coli*	-	This study
U115 R4	*E. coli*	-	This study
U115 R5	*E. coli*	-	This study
U115 R6	*E. coli*	-	This study
U115 R7	*E. coli*	-	This study
U115 R8	*E. coli*	-	This study
U115 R9	*E. coli*	-	This study
U115 R10	*E. coli*	-	This study
alb R1	*E. coli*	-	This study
alb R2	*E. coli*	-	This study
alb R3	*E. coli*	-	This study
alb R4	*E. coli*	-	This study
alb R5	*E. coli*	-	This study
alb R6	*E. coli*	-	This study
alb R7	*E. coli*	-	This study
alb R8	*E. coli*	-	This study
alb R9	*E. coli*	-	This study
XA23	*X. albilineans*	-	Rott et al., 1996
LS126	*X. albilineans*	KanR, StR	Rott et al., 1996

Phage	Reference or Source
T6	J. Wertz (Yale U)
U115	Kortright et al., 2020

KanR, Kanamycin resistance; StR, Streptomycin resistance; CGSC, Coli Genetic Stock Center
